# 5-(2-Methyl­phen­yl)-1,3,4-thia­diazol-2-amine

**DOI:** 10.1107/S1600536809011234

**Published:** 2009-04-02

**Authors:** Yao Wang, Xiang-Jun Kong, Rong Wan, Feng Han, Peng Wang

**Affiliations:** a Department of Applied Chemistry, College of Science, Nanjing University of Technology, No. 5 Xinmofan Road, Nanjing 210009, People’s Republic of China

## Abstract

The asymmetric unit of the title compound, C_9_H_9_N_3_S, contains two crystallographically independent mol­ecules, in which the thia­diazole and tolyl rings are oriented at dihedral angles of 32.25 (3) and 74.50 (3)°. An intra­molecular C—H⋯S inter­action results in the formation of a five-membered ring. In the crystal structure, inter­molecular N—H⋯N hydrogen bonds link the mol­ecules into chains along the *a* axis. A π–π contact between the thia­diazole rings [centroid–centroid distance = 3.910 (3) Å] may further stabilize the structure. There is also a weak C—H⋯π inter­action.

## Related literature

For the biological activity of 1,3,4-thiadiazole derivatives, see: Nakagawa *et al.* (1996 ▶); Wang *et al.* (1999 ▶). For a related structure, see: Han *et al.* (2007 ▶). For bond-length data, see: Allen *et al.* (1987 ▶).
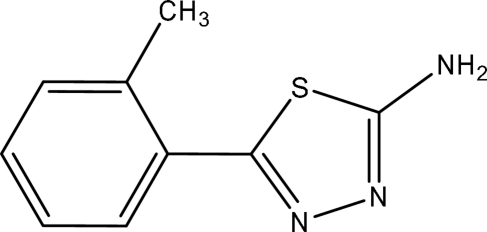


## Experimental

### 

#### Crystal data

C_9_H_9_N_3_S
*M* *_r_* = 191.26
Monoclinic, 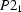

*a* = 10.792 (2) Å
*b* = 7.3400 (15) Å
*c* = 11.831 (2) Å
β = 96.15 (3)°
*V* = 931.8 (3) Å^3^
*Z* = 4
Mo *K*α radiation
μ = 0.30 mm^−1^
*T* = 294 K
0.20 × 0.10 × 0.10 mm

#### Data collection

Enraf–Nonius CAD-4 diffractometer
Absorption correction: ψ scan (North *et al.*, 1968 ▶) *T*_min_ = 0.942, *T*_max_ = 0.971
2190 measured reflections
2190 independent reflections
1620 reflections with *I* > 2σ(*I*)
*R*_int_ = 0.0000
3 standard reflections frequency: 120 min intensity decay: 1%

#### Refinement

*R*[*F* ^2^ > 2σ(*F* ^2^)] = 0.053
*wR*(*F* ^2^) = 0.162
*S* = 1.00
2190 reflections
237 parameters
1 restraint
H-atom parameters constrained
Δρ_max_ = 0.29 e Å^−3^
Δρ_min_ = −0.27 e Å^−3^
Absolute structure: Flack (1983 ▶), 113 Friedel pairs
Flack parameter: 0.04 (18)

### 

Data collection: *CAD-4 Software* (Enraf–Nonius, 1989 ▶); cell refinement: *CAD-4 Software* ; data reduction: *XCAD4* (Harms & Wocadlo, 1995 ▶); program(s) used to solve structure: *SHELXS97* (Sheldrick, 2008 ▶); program(s) used to refine structure: *SHELXL97* (Sheldrick, 2008 ▶); molecular graphics: *SHELXTL* (Sheldrick, 2008 ▶); software used to prepare material for publication: *SHELXL97*.

## Supplementary Material

Crystal structure: contains datablocks global, I. DOI: 10.1107/S1600536809011234/hk2633sup1.cif

Structure factors: contains datablocks I. DOI: 10.1107/S1600536809011234/hk2633Isup2.hkl

Additional supplementary materials:  crystallographic information; 3D view; checkCIF report

## Figures and Tables

**Table 1:** Hydrogen-bond geometry (Å, °)

*D*—H⋯*A*	*D*—H	H⋯*A*	*D*⋯*A*	*D*—H⋯*A*
N3*A*—H3*A*⋯N2*B*^i^	0.86	2.23	3.079 (8)	167
N3*A*—H3*B*⋯N1*B*^ii^	0.86	2.16	2.990 (6)	162
N3*B*—H6*B*⋯N2*A*^iii^	0.86	2.22	3.006 (8)	153
N3*B*—H6*C*⋯N1*A*	0.86	2.23	3.035 (6)	156
C6*A*—H6*A*⋯S1*A*	0.93	2.71	3.090 (8)	105
C1*B*—H10*A*⋯*Cg*3^iv^	0.96	2.89	3.623 (3)	134

Symmetry codes: (i) 
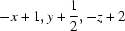
; (ii) 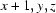; (iii) 
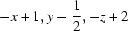
; (iv) 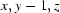.

**Fig. 1.**
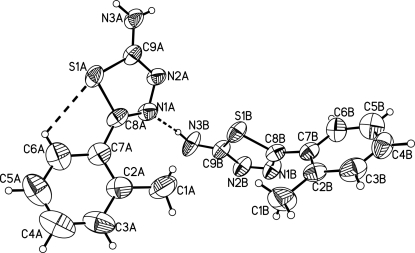
The molecular structure of the title molecule, with the atom-numbering scheme. Displacement ellipsoids are drawn at the 50% probability level. Hydrogen bonds are shown as dashed lines.

**Fig. 2.**
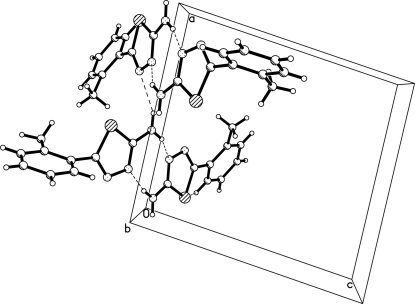
A partial packing diagram of the title compound. Hydrogen bonds are shown as dashed lines.

## supplementary crystallographic information

### Comment

1,3,4-Thiadiazole derivatives represent an interesting class of compounds possessing broad spectrum biological activities (Nakagawa *et al.*, 1996; Wang *et al.*, 1999). These compounds are known to exhibit diverse biological effects, such as insecticidal and fungicidal activities (Wang *et al.*, 1999). We are focused our synthetic and structural studies on 1,3,4 -thiadiazole derivatives and we have published the structure of 5-*m* -tolyl-1,3,4-thiadiazol-2-ylamine (Han *et al.*, 2007). We report herein the crystal structure of the title compound.

The asymmetric unit of the title compound contains two crystallographically independent molecules (Fig. 1), in which the bond lengths (Allen *et al.*, 1987) and angles are generally within normal ranges. Rings A (C2A-C7A), B (S1A/N1A/N2A/C8A/C9A) and C (C2B-C7B), D (S1B/N1B/N2B/C8B/C9B) are, of course, planar, and they are oriented at dihedral angles of A/B = 32.25 (3) and C/D = 74.50 (3) °. The intramolecular C-H···S interaction (Table 1) results in the formation of a five-membered ring E (S1A/C6A-C8A/H6A) adopting envelope conformation with S1A atom displaced by 0.813 (3) Å from the plane of the other ring atoms.

In the crystal structure, intra- and intermolecular N-H···N hydrogen bonds (Table 1) link the molecules into chains along the a axis (Fig. 2), in which they may be effective in the stabilization of the structure. The π-π contact between the thiadiazole rings, Cg1—Cg2^i^ [symmetry code: (i) x, 1 + y, -1 + z, where Cg1 and Cg2 are centroids of the rings B (S1A/N1A/N2A/C8A/C9A) and D (S1B/N1B/N2B/C8B/C9B), respectively] may further stabilize the structure, with centroid-centroid distance of 3.910 (3) Å. There also exists a weak C—H···π interaction (Table 1).

### Experimental

For the preparation of the title compound, 3-methyl-benzoic acid (5 mmol) and thiosemicarbazide (5 mmol) were added in toluene (50 ml), and kept in the oil bath at 363 K for 6 h. After cooling, the crude product precipitated and was filted. Crystals suitable for X-ray analysis were obtained by slow evaporation of an acetone solution.

### Refinement

H atoms were positioned geometrically, with N-H = 0.86 Å (for NH_2_) and C-H = 0.93 and 0.96 Å for aromatic and methyl H, respectively, and constrained to ride on their parent atoms, with U_iso_(H) = xU_eq_(C,N), where x = 1.5 for methyl H and x = 1.2 for all other H atoms.

### Crystal data

**Table:**

C_9_H_9_N_3_S	*F*(000) = 400
*M**_r_* = 191.26	*D*_x_ = 1.363 Mg m^−^^3^
Monoclinic, *P*2_1_	Melting point: 541 K
Hall symbol: P 2yb	Mo *K*α radiation, λ = 0.71073 Å
*a* = 10.792 (2) Å	Cell parameters from 25 reflections
*b* = 7.3400 (15) Å	θ = 10–13°
*c* = 11.831 (2) Å	µ = 0.30 mm^−^^1^
β = 96.15 (3)°	*T* = 294 K
*V* = 931.8 (3) Å^3^	Block, colorless
*Z* = 4	0.20 × 0.10 × 0.10 mm

### Data collection

**Table:**

Enraf–Nonius CAD-4 diffractometer	1620 reflections with *I* > 2σ(*I*)
Radiation source: fine-focus sealed tube	*R*_int_ = 0.0000
graphite	θ_max_ = 27.0°, θ_min_ = 1.7°
ω/2θ scans	*h* = −13→13
Absorption correction: ψ scan (North *et al.*, 1968)	*k* = 0→9
*T*_min_ = 0.942, *T*_max_ = 0.971	*l* = 0→15
2190 measured reflections	3 standard reflections every 120 min
2190 independent reflections	intensity decay: 1%

### Refinement

**Table:**

Refinement on *F*^2^	Secondary atom site location: difference Fourier map
Least-squares matrix: full	Hydrogen site location: inferred from neighbouring sites
*R*[*F*^2^ > 2σ(*F*^2^)] = 0.053	H-atom parameters constrained
*wR*(*F*^2^) = 0.162	*w* = 1/[σ^2^(*F*_o_^2^) + (0.1*P*)^2^ + 0.06*P*] where *P* = (*F*_o_^2^ + 2*F*_c_^2^)/3
*S* = 1.00	(Δ/σ)_max_ < 0.001
2190 reflections	Δρ_max_ = 0.29 e Å^−^^3^
237 parameters	Δρ_min_ = −0.27 e Å^−^^3^
1 restraint	Absolute structure: Flack (1983), 113 Friedel pairs
Primary atom site location: structure-invariant direct methods	Flack parameter: 0.04 (18)

### Special details

**Table:**

Geometry. All e.s.d.'s (except the e.s.d. in the dihedral angle between two l.s. planes) are estimated using the full covariance matrix. The cell e.s.d.'s are taken into account individually in the estimation of e.s.d.'s in distances, angles and torsion angles; correlations between e.s.d.'s in cell parameters are only used when they are defined by crystal symmetry. An approximate (isotropic) treatment of cell e.s.d.'s is used for estimating e.s.d.'s involving l.s. planes.
Refinement. Refinement of *F*^2^ against ALL reflections. The weighted *R*-factor *wR* and goodness of fit *S* are based on *F*^2^, conventional *R*-factors *R* are based on *F*, with *F* set to zero for negative *F*^2^. The threshold expression of *F*^2^ > σ(*F*^2^) is used only for calculating *R*-factors(gt) *etc*. and is not relevant to the choice of reflections for refinement. *R*-factors based on *F*^2^ are statistically about twice as large as those based on *F*, and *R*- factors based on ALL data will be even larger.

### Fractional atomic coordinates and isotropic or equivalent isotropic displacement parameters (Å^2^)

**Table:**

	*x*	*y*	*z*	*U*_iso_*/*U*_eq_	
S1A	0.87687 (12)	0.61482 (19)	0.83012 (11)	0.0525 (4)	
N1A	0.6530 (4)	0.6359 (8)	0.8777 (4)	0.0504 (12)	
N2A	0.7123 (4)	0.7860 (7)	0.9302 (4)	0.0506 (12)	
N3A	0.9083 (4)	0.9167 (8)	0.9573 (4)	0.0572 (13)	
H3A	0.8827	1.0019	0.9990	0.069*	
H3B	0.9850	0.9135	0.9439	0.069*	
C1A	0.4713 (6)	0.4830 (15)	0.6920 (7)	0.102 (3)	
H1B	0.4032	0.4467	0.6378	0.153*	
H1C	0.5046	0.5967	0.6688	0.153*	
H1D	0.4422	0.4970	0.7654	0.153*	
C2A	0.5735 (6)	0.3366 (11)	0.6984 (5)	0.0719 (19)	
C3A	0.5524 (9)	0.1754 (12)	0.6397 (6)	0.089 (3)	
H3C	0.4748	0.1541	0.5997	0.107*	
C4A	0.6461 (11)	0.0427 (13)	0.6393 (7)	0.101 (3)	
H4A	0.6297	−0.0665	0.6008	0.121*	
C5A	0.7622 (10)	0.0745 (13)	0.6961 (6)	0.099 (3)	
H5A	0.8256	−0.0109	0.6943	0.118*	
C6A	0.7826 (8)	0.2317 (11)	0.7545 (5)	0.078 (2)	
H6A	0.8605	0.2506	0.7943	0.094*	
C7A	0.6914 (6)	0.3683 (10)	0.7579 (4)	0.0584 (15)	
C8A	0.7246 (5)	0.5356 (9)	0.8216 (4)	0.0498 (14)	
C9A	0.8294 (5)	0.7902 (9)	0.9137 (4)	0.0480 (13)	
S1B	0.37988 (11)	0.8934 (2)	0.83030 (11)	0.0573 (5)	
N1B	0.1506 (4)	0.8716 (9)	0.8594 (4)	0.0568 (13)	
N2B	0.2089 (4)	0.7424 (9)	0.9293 (4)	0.0584 (13)	
N3B	0.4089 (4)	0.6193 (10)	0.9831 (4)	0.0746 (18)	
H6B	0.3816	0.5427	1.0295	0.090*	
H6C	0.4870	0.6226	0.9745	0.090*	
C1B	0.2175 (8)	0.8875 (11)	0.5560 (5)	0.082 (2)	
H10A	0.2077	0.8889	0.4744	0.124*	
H10B	0.1631	0.7973	0.5827	0.124*	
H10C	0.3023	0.8586	0.5829	0.124*	
C2B	0.1846 (5)	1.0742 (9)	0.6006 (4)	0.0532 (13)	
C3B	0.1501 (6)	1.2157 (11)	0.5268 (5)	0.0688 (17)	
H12A	0.1477	1.1966	0.4489	0.083*	
C4B	0.1192 (5)	1.3853 (10)	0.5666 (5)	0.0660 (16)	
H13A	0.0960	1.4783	0.5152	0.079*	
C5B	0.1226 (6)	1.4175 (13)	0.6825 (6)	0.076 (2)	
H14A	0.1014	1.5312	0.7093	0.092*	
C6B	0.1574 (6)	1.2795 (9)	0.7564 (5)	0.0616 (16)	
H15A	0.1616	1.3012	0.8342	0.074*	
C7B	0.1866 (4)	1.1078 (9)	0.7184 (4)	0.0466 (12)	
C8B	0.2252 (5)	0.9641 (9)	0.8013 (4)	0.0469 (13)	
C9B	0.3289 (5)	0.7347 (9)	0.9235 (4)	0.0496 (14)	

### Atomic displacement parameters (Å^2^)

**Table:**

	*U*^11^	*U*^22^	*U*^33^	*U*^12^	*U*^13^	*U*^23^
S1A	0.0373 (7)	0.0732 (9)	0.0484 (7)	0.0080 (7)	0.0115 (5)	−0.0080 (7)
N1A	0.040 (2)	0.066 (3)	0.048 (2)	−0.007 (2)	0.0155 (18)	−0.010 (2)
N2A	0.038 (2)	0.065 (3)	0.051 (3)	−0.005 (2)	0.012 (2)	−0.018 (2)
N3A	0.039 (2)	0.075 (3)	0.060 (3)	0.002 (3)	0.014 (2)	−0.021 (3)
C1A	0.057 (4)	0.148 (8)	0.098 (5)	−0.003 (5)	−0.004 (4)	−0.041 (6)
C2A	0.063 (4)	0.097 (5)	0.060 (3)	−0.016 (4)	0.022 (3)	−0.019 (4)
C3A	0.106 (6)	0.094 (6)	0.072 (5)	−0.041 (5)	0.031 (4)	−0.025 (4)
C4A	0.165 (9)	0.081 (5)	0.062 (4)	−0.044 (6)	0.042 (6)	−0.003 (4)
C5A	0.159 (9)	0.086 (5)	0.051 (4)	0.022 (6)	0.016 (5)	0.003 (4)
C6A	0.104 (5)	0.083 (5)	0.048 (3)	0.013 (4)	0.008 (3)	0.007 (4)
C7A	0.061 (3)	0.076 (4)	0.039 (3)	−0.005 (3)	0.015 (2)	0.006 (3)
C8A	0.039 (3)	0.078 (4)	0.032 (2)	−0.006 (3)	0.002 (2)	0.001 (3)
C9A	0.039 (3)	0.069 (3)	0.036 (2)	0.003 (3)	0.007 (2)	−0.001 (3)
S1B	0.0337 (7)	0.0842 (11)	0.0549 (8)	−0.0021 (8)	0.0086 (6)	0.0203 (9)
N1B	0.038 (2)	0.086 (4)	0.048 (2)	0.002 (3)	0.0128 (18)	0.018 (3)
N2B	0.036 (2)	0.089 (4)	0.053 (3)	0.000 (3)	0.017 (2)	0.013 (3)
N3B	0.035 (2)	0.115 (5)	0.075 (3)	0.013 (3)	0.012 (2)	0.047 (4)
C1B	0.114 (6)	0.085 (5)	0.047 (3)	−0.002 (5)	0.004 (3)	−0.023 (4)
C2B	0.053 (3)	0.066 (3)	0.040 (2)	−0.011 (3)	0.002 (2)	−0.005 (3)
C3B	0.073 (4)	0.092 (5)	0.041 (3)	−0.014 (4)	0.002 (3)	0.005 (3)
C4B	0.053 (3)	0.081 (4)	0.065 (4)	0.005 (3)	0.009 (3)	0.021 (4)
C5B	0.071 (4)	0.091 (5)	0.071 (4)	0.005 (4)	0.029 (3)	0.000 (4)
C6B	0.064 (4)	0.072 (4)	0.052 (3)	0.014 (3)	0.023 (3)	0.000 (3)
C7B	0.037 (2)	0.062 (3)	0.041 (3)	−0.008 (3)	0.006 (2)	0.005 (3)
C8B	0.037 (3)	0.066 (3)	0.039 (3)	−0.006 (3)	0.010 (2)	−0.001 (3)
C9B	0.037 (3)	0.073 (4)	0.040 (3)	−0.002 (3)	0.012 (2)	0.010 (3)

### Geometric parameters (Å, °)

**Table:**

S1A—C9A	1.734 (6)	S1B—C9B	1.733 (6)
S1A—C8A	1.736 (5)	S1B—C8B	1.747 (5)
N1A—C8A	1.300 (7)	N1B—C8B	1.304 (7)
N1A—N2A	1.387 (7)	N1B—N2B	1.366 (8)
N2A—C9A	1.299 (6)	N2B—C9B	1.306 (6)
N3A—C9A	1.326 (7)	N3B—C9B	1.352 (8)
N3A—H3A	0.8600	N3B—H6B	0.8600
N3A—H3B	0.8600	N3B—H6C	0.8600
C1A—C2A	1.536 (11)	C1B—C2B	1.524 (9)
C1A—H1B	0.9600	C1B—H10A	0.9600
C1A—H1C	0.9600	C1B—H10B	0.9600
C1A—H1D	0.9600	C1B—H10C	0.9600
C2A—C3A	1.379 (10)	C2B—C3B	1.382 (9)
C2A—C7A	1.406 (9)	C2B—C7B	1.413 (7)
C3A—C4A	1.404 (13)	C3B—C4B	1.384 (10)
C3A—H3C	0.9300	C3B—H12A	0.9300
C4A—C5A	1.377 (12)	C4B—C5B	1.388 (9)
C4A—H4A	0.9300	C4B—H13A	0.9300
C5A—C6A	1.351 (12)	C5B—C6B	1.365 (10)
C5A—H5A	0.9300	C5B—H14A	0.9300
C6A—C7A	1.408 (10)	C6B—C7B	1.386 (8)
C6A—H6A	0.9300	C6B—H15A	0.9300
C7A—C8A	1.465 (9)	C7B—C8B	1.470 (8)
			
C9A—S1A—C8A	86.9 (3)	C9B—S1B—C8B	87.9 (3)
C8A—N1A—N2A	114.1 (4)	C8B—N1B—N2B	114.2 (4)
C9A—N2A—N1A	111.2 (5)	C9B—N2B—N1B	113.2 (5)
C9A—N3A—H3A	120.0	C9B—N3B—H6B	120.0
C9A—N3A—H3B	120.0	C9B—N3B—H6C	120.0
H3A—N3A—H3B	120.0	H6B—N3B—H6C	120.0
C2A—C1A—H1B	109.5	C2B—C1B—H10A	109.5
C2A—C1A—H1C	109.5	C2B—C1B—H10B	109.5
H1B—C1A—H1C	109.5	H10A—C1B—H10B	109.5
C2A—C1A—H1D	109.5	C2B—C1B—H10C	109.5
H1B—C1A—H1D	109.5	H10A—C1B—H10C	109.5
H1C—C1A—H1D	109.5	H10B—C1B—H10C	109.5
C3A—C2A—C7A	119.1 (8)	C3B—C2B—C7B	117.8 (6)
C3A—C2A—C1A	119.8 (7)	C3B—C2B—C1B	121.0 (5)
C7A—C2A—C1A	121.0 (6)	C7B—C2B—C1B	121.2 (5)
C2A—C3A—C4A	121.1 (8)	C2B—C3B—C4B	121.3 (6)
C2A—C3A—H3C	119.5	C2B—C3B—H12A	119.3
C4A—C3A—H3C	119.5	C4B—C3B—H12A	119.3
C5A—C4A—C3A	119.9 (8)	C3B—C4B—C5B	120.5 (7)
C5A—C4A—H4A	120.0	C3B—C4B—H13A	119.8
C3A—C4A—H4A	120.0	C5B—C4B—H13A	119.8
C6A—C5A—C4A	119.0 (9)	C6B—C5B—C4B	118.9 (7)
C6A—C5A—H5A	120.5	C6B—C5B—H14A	120.5
C4A—C5A—H5A	120.5	C4B—C5B—H14A	120.5
C5A—C6A—C7A	123.2 (8)	C5B—C6B—C7B	121.5 (5)
C5A—C6A—H6A	118.4	C5B—C6B—H15A	119.2
C7A—C6A—H6A	118.4	C7B—C6B—H15A	119.2
C2A—C7A—C6A	117.7 (7)	C6B—C7B—C2B	119.9 (5)
C2A—C7A—C8A	123.6 (6)	C6B—C7B—C8B	119.6 (5)
C6A—C7A—C8A	118.6 (6)	C2B—C7B—C8B	120.4 (6)
N1A—C8A—C7A	127.7 (5)	N1B—C8B—C7B	125.4 (5)
N1A—C8A—S1A	113.0 (5)	N1B—C8B—S1B	111.9 (4)
C7A—C8A—S1A	119.2 (4)	C7B—C8B—S1B	122.7 (4)
N2A—C9A—N3A	123.5 (5)	N2B—C9B—N3B	125.5 (5)
N2A—C9A—S1A	114.8 (5)	N2B—C9B—S1B	112.9 (4)
N3A—C9A—S1A	121.7 (4)	N3B—C9B—S1B	121.6 (4)
			
C8A—N1A—N2A—C9A	−1.8 (7)	C8B—N1B—N2B—C9B	−0.8 (8)
C7A—C2A—C3A—C4A	0.4 (10)	C7B—C2B—C3B—C4B	0.3 (9)
C1A—C2A—C3A—C4A	176.9 (7)	C1B—C2B—C3B—C4B	179.5 (6)
C2A—C3A—C4A—C5A	−1.5 (11)	C2B—C3B—C4B—C5B	0.3 (10)
C3A—C4A—C5A—C6A	2.1 (11)	C3B—C4B—C5B—C6B	0.3 (10)
C4A—C5A—C6A—C7A	−1.8 (11)	C4B—C5B—C6B—C7B	−1.4 (10)
C3A—C2A—C7A—C6A	−0.1 (9)	C5B—C6B—C7B—C2B	2.0 (9)
C1A—C2A—C7A—C6A	−176.4 (6)	C5B—C6B—C7B—C8B	179.2 (5)
C3A—C2A—C7A—C8A	179.1 (6)	C3B—C2B—C7B—C6B	−1.5 (8)
C1A—C2A—C7A—C8A	2.7 (9)	C1B—C2B—C7B—C6B	179.4 (6)
C5A—C6A—C7A—C2A	0.8 (10)	C3B—C2B—C7B—C8B	−178.6 (5)
C5A—C6A—C7A—C8A	−178.4 (6)	C1B—C2B—C7B—C8B	2.2 (8)
N2A—N1A—C8A—C7A	179.5 (5)	N2B—N1B—C8B—C7B	178.6 (6)
N2A—N1A—C8A—S1A	1.3 (6)	N2B—N1B—C8B—S1B	0.3 (7)
C2A—C7A—C8A—N1A	34.2 (9)	C6B—C7B—C8B—N1B	77.1 (8)
C6A—C7A—C8A—N1A	−146.6 (6)	C2B—C7B—C8B—N1B	−105.7 (7)
C2A—C7A—C8A—S1A	−147.7 (5)	C6B—C7B—C8B—S1B	−104.7 (6)
C6A—C7A—C8A—S1A	31.4 (7)	C2B—C7B—C8B—S1B	72.5 (7)
C9A—S1A—C8A—N1A	−0.4 (5)	C9B—S1B—C8B—N1B	0.2 (5)
C9A—S1A—C8A—C7A	−178.7 (5)	C9B—S1B—C8B—C7B	−178.2 (5)
N1A—N2A—C9A—N3A	−178.1 (5)	N1B—N2B—C9B—N3B	−179.8 (6)
N1A—N2A—C9A—S1A	1.4 (6)	N1B—N2B—C9B—S1B	1.0 (7)
C8A—S1A—C9A—N2A	−0.6 (5)	C8B—S1B—C9B—N2B	−0.7 (5)
C8A—S1A—C9A—N3A	178.9 (5)	C8B—S1B—C9B—N3B	−179.9 (6)

### Hydrogen-bond geometry (Å, °)

**Table:**

*D*—H···*A*	*D*—H	H···*A*	*D*···*A*	*D*—H···*A*
N3A—H3A···N2B^i^	0.86	2.23	3.079 (8)	167
N3A—H3B···N1B^ii^	0.86	2.16	2.990 (6)	162
N3B—H6B···N2A^iii^	0.86	2.22	3.006 (8)	153
N3B—H6C···N1A	0.86	2.23	3.035 (6)	156
C6A—H6A···S1A	0.93	2.71	3.090 (8)	105
C1B—H10A···Cg3^iv^	0.96	2.89	3.623 (3)	134

Symmetry codes: (i) −*x*+1, *y*+1/2, −*z*+2; (ii) *x*+1, *y*, *z*; (iii) −*x*+1, *y*−1/2, −*z*+2; (iv) *x*, *y*−1, *z*.

## References

[bb1] Allen, F. H., Kennard, O., Watson, D. G., Brammer, L., Orpen, A. G. & Taylor, R. (1987). *J. Chem. Soc. Perkin Trans. 2*, pp. S1–19.

[bb2] Enraf–Nonius (1989). *CAD-4 Software* Enraf–Nonius, Delft, The Netherlands.

[bb3] Flack, H. D. (1983). *Acta Cryst.* A**39**, 876–881.

[bb4] Han, F., Wan, R., Wu, W.-Y., Zhang, J.-J. & Wang, J.-T. (2007). *Acta Cryst.* E**63**, o717–o718.

[bb5] Harms, K. & Wocadlo, S. (1995). *XCAD4* University of Marburg, Germany.

[bb6] Nakagawa, Y., Nishimura, K., Izumi, K., Kinoshita, K., Kimura, T. & Kurihara, N. (1996). *J. Pestic. Sci.* **21**, 195–201.

[bb7] North, A. C. T., Phillips, D. C. & Mathews, F. S. (1968). *Acta Cryst.* A**24**, 351–359.

[bb8] Sheldrick, G. M. (2008). *Acta Cryst.* A**64**, 112–122. 10.1107/S0108767307043930 18156677

[bb9] Wang, Y. G., Cao, L., Yan, J., Ye, W. F., Zhou, Q. C. & Lu, B. X. (1999). *Chem. J. Chin. Univ.* **20**, 1903–1905.

